# Long-term evaluation of the efficacy and safety of Nd:YAG laser vitreolysis for symptomatic vitreous foaters

**DOI:** 10.5935/0004-2749.2021-0395

**Published:** 2022-09-06

**Authors:** Guilherme M. Nunes, Gustavo D. Ludwig, Henrique Gemelli, Márgara Zanotele, Pedro D. Serracarbassa

**Affiliations:** 1 Ophthalmology Department, Hospital do Servidor Público Estadual, São Paulo, SP, Brazil

**Keywords:** Laser therapy, Lasers, solid-state, Vitrectomy, Vitreous body, Vitreoretinal surgery, Visual acuity, Eye diseases, Quality of life, Surveys and questionnaires, Terapia a laser, Lasers de estado sólido, Vitrectomia, Corpo vítreo, Cirurgia vitreorretiniana, Acuidade visual, Doenças oculares, Qualidade de vida, Inquéritos e questionários

## Abstract

**Purpose:**

This study aimed to evaluate the long-term safety and efficacy of neodymium-doped yttrium aluminum garnet (Nd:YAG) vitreolysis for symptomatic vitreous floaters as it remains a controversial procedure due to insufficient robust evidence in the literature for the maintenance of the results and absence of adverse effects.

**Methods:**

This is an observational extension to the previously presented prospective, randomized, double-blind clinical trial. Eight of thirteen subjects who underwent vitreolysis with YAG laser returned for a late reevaluation, 18 months after the procedure, to evaluate the efficacy and safety of the procedure.

**Results:**

All patients maintained the improvement in symptomatology noted after the procedure, with 25% showing complete improvement and a similar proportion (37.5%) reporting significant or partial improvement. Objective improvement in opacity was similar to that found at 6 months follow-up. The NEI-VFQ 25 quality of life questionnaire showed no statistically significant difference in responses between the 6^th^ and 18^th^ month. No adverse effects were noted on clinical examination or reported by patients.

**Conclusion:**

Vitreolysis efficacy observed at 6 months of follow-up was maintained until the eighteenth month, with all patients reporting improvement from the pre-procedure state. No late adverse effects were noted. A larger randomized clinical trial is needed to confirm the safety of the procedure.

## Abstract (pt)

**Objetivos:**

Avaliar a segurança e eficácia a longo prazo da vitreólise com Nd:YAG laser para moscas volantes sintomáticas, uma vez que permanece como um procedimento controverso devido a falta de evidência científica robusta sobre a manutenção dos resultados e ocorrência de efeitos adversos.

**Métodos:**

Este estudo é uma extensão observacional de um ensaio clínico prospectivo, randomizado, duplo cego, previamente publicado. Oito de treze pacientes que foram submetidos a vitreólise com YAG laser foram acompanhados para uma reavaliação tardia, dezoito meses após o procedimento, para avaliar a eficácia e segurança do procedimento.

**Resultados:**

Todos os pacientes mantiveram a melhora na sintomatologia notada ao final do procedimento original, com 25% dos casos apresentando melhora completa, e uma proporção semelhante (37,5%) demonstrando melhora significativa ou parcial. A melhora objetiva na opacidade foi similar ao achado no seguimento original de 6 meses. O questionário de qualidade de vida NEI-VFQ 25 não demonstrou diferença estatisticamente significativa nas respostas entre o sexto e o décimo oitavo mês de acompanhamento. Nenhum efeito adverso foi notado no exame clínico ou reportado pelos pacientes.

**Conclusão:**

A eficácia da vitreólise observada ao sexto mês do acompanhamento foi mantida até o décimo oitavo mês, com todos os pacientes notando algum grau de melhora quando comparado ao estado pré procedimento. Nenhum efeito adverso tardio foi notado. Um ensaio clínico randomizado maior é necessário para confirmar a segurança do procedimento.

## INTRODUCTION

Vitreous floaters result from posterior vitreous detachment (PVD) and is clinically perceived as vitreous opacities that present as moving dark spots in the vision^(1,2)^.

It is one of the most common complaints to ophthalmologists. In the majority of patients, this condition is not usually symptomatic, mainly when related to opacities outside the visual axis or after a certain period of neuroadaptation. However, in a proportion of individuals, mainly detailers, individuals with myopia or pseudophakia, vitreous floaters can be extremely bothersome, interfering with perception and daily visual comfort, leading to psychological and physical exhaustion^(3,4,5)^.

Some management options in the face of symptomatic vitreous floaters are patient observation and orientation, pars plana vitrectomy, and laser vitreolysis.

To prevent the complications of vitrectomy, such as retinal breaks, cataract development, retinal detachment, choroidal hemorrhage, and vitreoretinal proliferation, vitreolysis with neodymium-doped yttrium aluminum garnet (Nd:YAG) laser has become an alternative treatment. The mechanism of laser vitreolysis is photodisruption of vitreous aggregates, causing reduction of opacity and displacement off the visual axis^(6,7,8,9,10)^.

Vitreolysis with Nd:YAG laser has lower financial cost to the patient and to the health system and lower demand of time as it does not require hospital admission and leads to lower emotional distress of the patient because of the shorter and less invasive process.

In our previous study, a randomized clinical trial published by Ludwig et al. in 2020, the Nd:YAG laser was applied in symptomatic patients for vitreolysis and showed a good safety profile and improvement in the symptomatology of vitreous opacities. A complete or significant improvement of vitreous floater-related symptomatology was demonstrated in 75% of patients. No patients who underwent laser had significant adverse effects, such as retinal ruptures, macular edema, or macular hole^(11)^.

Other studies, such as the study by Shah et al., have obtained compatible results. In this study, a series of 36 eyes submitted to vitreolysis with Nd:YAG laser was analyzed. It showed an improvement of 54% in the symptoms of patients undergoing the procedure without the occurrence of significant side effects^(12)^. This result is corroborated by Souza et al., who reported an objective improvement of 93.7% and subjective improvement of 46.1% after a single laser session^(13)^.

However, vitreolysis with YAG laser is still a controversial treatment due to the lack of robust evidence in the literature regarding its safety and lack of long-term follow-up^(12)^.

There are several complications related to the method described in the literature, including prolonged increase in intraocular pressure, development of cataract, intraocular lens damage, posterior capsule defects, retinal rupture, retinal hemorrhage, and retinal detachment^(14,15)^. However, the vast majority of studies are reports on isolated cases, so the real risk and complication rate of the procedure is unknown^(16)^.

This study aimed to follow-up patients from the original clinical trial who underwent vitreolysis with YAG laser, evaluating the efficacy of the procedure by measuring the maintenance of long-term benefit and assessing the risk of late complications of the procedure.

## METHODS

### Population

This study evaluated the efficacy and long-term safety of another previously presented study: a randomized, controlled, masked, double-blind clinical trial conducted at a single hospital center in São Paulo, Brazil. The initial clinical trial included a total of 24 patients at the *Hospital do Servidor Público Estadual de São Paulo*. They were randomized and divided into two groups: YAG laser intervention (13 patients) or control and simulated procedure (11 patients). The Ethics Committee of the São Paulo State Public Servant Hospital approved the off-label use of the YAG laser for vitreolysis in this study (reference number: 2.755.274). The study was performed in accordance with the principles of the Declaration of Helsinki and registered in the Brazilian Registry of Clinical Trials under code RBR-2jq3v. The initial planned and submitted follow-up was six months.

All procedures were performed by the same physician in only one laser session. A Volk Singh Mid-Vitreous lens was positioned, and vitreolysis was performed using the VISULAS YAG III (Zeiss) device. The energy was initially fixed at 4 mJ and slowly increased to a level, at which the physician observed the photodisruption of the opacity and formation of gas bubbles. The patients had an average number of 144.7 ± 46.1 laser shots, with a mean energy of 6.1 ± 1.4 mJ per pulse.

Long-term follow-up included 13 patients treated with vitreolysis with YAG laser in a single hospital center in São Paulo, Brazil, who were examined between July 2018 and September 2020. Five patients missed the follow-up for the following reasons: two did not answer repeated phone calls, and three refused to participate due to the COVID-19 pandemic.

All patients were followed up for 18 months, with clinical examinations performed at the following post-procedure periods: week 1 and month 1, 3, 6, and 18. The primary outcomes, measured at month 6 and 18, were as follows: 10-point visual disturbance score as described by Singh^(17)^, four-level qualitative scale as described by Delaney et al.^(8)^, contrast sensitivity measured with the Pelli-Robson table, and the National Eye Institute Visual Functioning Questionnaire 25 (NEI-VFQ-25) adapted to Portuguese^(18)^. Of these, the NEI-VFQ-25 is the only validated method. Secondary endpoints included objective change in vitreous opacities based on masked retinography grading, visual acuity with best correction, intraocular pressure (IOP) change, and adverse event assessment.

### Statistical analysis

The data were organized and recorded in a database in Microsoft Office Excel 2007® program with double entry. Statistical analysis was performed using Stata® 11 SE.

The normality of the variables was tested by the Shapiro-Wilk test. The evaluated variables were presented in tables with absolute and relative frequency distribution. Associations were analyzed using Pearson’s chi-square test or Fisher’s exact test, when necessary.

Statistical significance of the differences in means between the quantitative variables was verified using the paired and unpaired Student’s t-test. The differences in variances were verified by analysis of variance with repeated measures, which was used to evaluate the different times within a group. All analyses were performed at a 5% significance level, and the results were considered statistically significant when the p-value was <0.05, always considering two-tailed alternative hypotheses.

## RESULTS

### Patient data

Thirteen eyes of 13 patients were included in the study. Five patients were lost to follow-up and excluded from the study. The mean age of the patients was 60 years, with a standard deviation of 7.7, ranging from 48 to 72 years. Most patients were female (75%) and had the right eye as the most symptomatic (75%). All patients were phakic, had a mean complaint time of 29 months, and rated 6.2 on the 0-10 symptom scale.

### Subjective and objective improvement

All patients who underwent the procedure reported symptom improvement. Most patients at the sixth month of follow-up showed a significant subjective improvement (50%), followed by complete improvement (25%) and partial improvement (25%). At the eighteenth month, the patients showed a similar proportion of partial and significant improvement (37.5%).

In the objective assessment by the blinded evaluator, at the sixth month of follow-up, a similar proportion of patients showed significant improvement (50%) and partial improvement (50%). This proportion was maintained at the eighteenth month.

### Intraocular pressure

The study patients started treatment with a mean IOP of 13.0 ± 3.7 mmHg. At the sixth month, they had a mean IOP of 14.4 ± 3.5 mmHg. There was no statistically significant difference between IOP measurements (p=0.841). The last IOP measurement (15.1 ± 2.7 mmHg), obtained at 18 months, also showed no statistically significant difference (p=0.963).

### NEI-VFQ 25

The intervention group reported a significantly better general vision (75.8 versus 59.2; p=0.037). A significant difference in mental health (p=0.048) was observed when comparing the sixth month values of the intervention (84.3) and control groups (70.3).

The answers given to the questionnaire from the initial time point at the 6^th^ and 18^th^ months of follow-up were compared, with no statistically significant difference observed.

### Subjective perception 0-10 scale

The patients in the study started with a subjective perception of symptomatology of 6.2 ± 1.0. At the sixth month, they presented a mean perception of 2.5 ± 2.4, representing a statistically significant difference (p=0.001). At 18 months, they had a subjective perception of 2.4 ± 2.3, maintaining the difference with statistical significance in relation to the initial evaluation.

### Adverse effects

No retinal detachment, retinal tear, uveitis, cystoid macular edema, macular hole, or other significant adverse effects were identified in the study. Three patients (37.5%) reported temporary blurring of vision after the procedure, with spontaneous resolution within the first day.

### Additional YAG laser treatment

Only one patient required and desired an additional session for retreatment with YAG laser vitreolysis. The patient is still being followed up for evaluation of symptomatology improvement and retreatment safety profile.

## DISCUSSION

The present study demonstrated expressive and consistent improvement in the symptoms of vitreous floaters after a single laser session, with 62.5% of treated patients reporting significant or complete improvement, even after 18 months of the procedure. However, this finding was slightly lower without statistical significance compared to that found at the sixth month of follow-up, with 75% of patients reporting this improvement^(11)^. These data are in agreement with the findings by Shah et al. who obtained 50% significant or complete improvement in a similar study on 34 patients at the end of a 2- or 3-year follow-up^(19)^.

Other comparative data were consistent with the findings at the sixth month of follow-up, such as the 0-10-point symptomatology scale, objective assessment of improvement of the appearance of opacity, and responses to the NEI-VFQ 25 quality of life questionnaire. None of the evaluated parameters showed a statistically significant difference between the sixth and eighteenth months. This suggests the durability of the long-term effects of vitreolysis with YAG laser and is corroborated by other long-term studies^(19)^.

The improvement in mental health found in the original study is interesting, especially considering that some patients who are bothered by floaters tend to have a higher anxiety psychological profile. This finding is supported by the study by Shah et al. in their long-term follow-up^(19)^.

In this study, follow-up was performed referring to only a single laser session. In the long-term study conducted by Shah et al., a second vitreolysis session was performed in the sixth month of follow-up, which showed additional improvement of 17.8% in the symptomatology related to floaters but without statistical relevance^(19)^. In our case series, two patients wished to undergo a new vitreolysis session: one in the same eye because he reported a slight worsening of the perception of opacity, and the other patient in the contralateral eye, which also presented floaters. A standardized follow-up of these patients has not yet been performed, and it is not possible to evaluate any further improvement or safety of a new procedure. Thus, the benefit of multiple sessions for this purpose should be re-evaluated in future studies, with a larger number of patients and more accurate criteria for retreatment.

There were no clinically significant adverse effects, such as retinal breaks, retinal detachment, cystoid macular edema, macular hole, uveitis, glaucoma, and cataract during the entire follow-up of these patients. This is in agreement with some other studies^(8,13,17)^.

Meanwhile, in the study by Shah et al., three late retinal tears were noted, which manifested between 1.4 and 2.8 years after the procedure. All ruptures were asymptomatic and detected during the clinical examination. This highlights the need for long-term follow-up of these patients and thorough clinical examination to confirm the safety of the procedure and importance of patient education about alarm symptoms. However, since there was no follow-up of a control group, it is not possible that these late retinal ruptures are related to the treatment performed or if it would already be a risk inherent to these eyes^(19)^.

Given a still uncertain safety profile due to the lack of robust evidence and well-designed studies, the selection of patients who will undergo vitreolysis is extremely important to reduce the risk of complications and maximize individual satisfaction with the procedure. Priority should be given to patients with single opacities that are a reasonable distance from the lens and retina. If the patient is having photopsias or a change in the floater pattern, suggestive of recent PVD, observation remains the best option^(20)^. It is also important to guide the patient well and inform them that multiple laser sessions may be required and that there is a chance that the treatment may not completely resolve the symptoms. All these aspects maximize the patient’s satisfaction with the procedure performed.

Some limitations are inherent to this study, including the small number of patients and limited follow-up. The small number of patients prevents the identification of potential rarer complications. The loss to follow-up of a considerable percentage of the initial participants may have led to a selection bias. This evaluation was performed from only one laser session; however, in a real-life scenario, more sessions may be required for complete resolution of opacities, which would theoretically further increase its effectiveness.

Another limitation is that only vitreous opacities associated with PVD and Weiss ring were treated, making it impossible to infer that these results would be replicable to other types of vitreous opacities.

Therefore, a large, randomized, controlled study with a long-term follow-up is needed to determine the real risks and benefits of vitreolysis with YAG laser, comparing this procedure with vitrectomy alone and with only observation and follow-up of cases^(21)^.

This study suggests that vitreolysis with Nd:YAG laser is effective and improves visual outcomes subjectively and objectively, without clinically relevant adverse effects in an 18-month follow-up period. It is proposed that this procedure may be indicated for patients presenting with visual disturbances secondary to clinically confirmed vitreous opacity and complete PVD confirmed by ultrasonography (B-scan).
